# Iliac vein variation in the sacral promontory on three-dimensional computed tomography angiography: a prospective observational study before laparoscopic sacrocolpopexy

**DOI:** 10.1007/s00192-023-05681-4

**Published:** 2023-11-24

**Authors:** Hirotaka Sato, Miki Kurita, Takehiro Kato, Hirokazu Abe, Shota Otsuka, Sachiyuki Tsukada

**Affiliations:** 1 Department of Urology, Hokusuikai Kinen Hospital, Ibaraki, Japan; 2 Department of Radiology, Hokusuikai Kinen Hospital, Ibaraki, Japan; 3 Department of surgery, Toyota Kosei Hospital, Aichi, Japan; 4 Department of Urology, Kameda Medical Center, Chiba, Japan; 5 Department of Orthopedics, Hokusuikai Kinen Hospital, Ibaraki, Japan

**Keywords:** Computed tomography, Iliac vessels, Pelvic organ prolapse, Prolapse, Sacral promontory, Sacrocolpopexy

## Abstract

**Introduction and Hypothesis:**

Venous injury may occur during exposure of the anterior longitudinal ligament at the anterior sacral promontory (SP). We aimed to quantitatively measure the extent of the vascular window (VW) in front of the SP in patients with internal iliac vein (IIV) variations using preoperative three-dimensional computed tomography angiography (3DCTA). We hypothesized that patients with IIV variations would have a narrow VW.

**Methods:**

This prospective observational study included patients scheduled for laparoscopic sacrocolpopexy (LSC) between July 2022 and April 2023 who underwent preoperative 3DCTA. The primary endpoint was the VW measurement in the standard and variant IIV groups using 3DCTA before LSC. The secondary endpoint was the difference between the two IIV groups adjusted for age, body mass index, hypertension, and diabetes using an analysis of covariance (ANCOVA) model. Multiple regression analysis was performed to analyze the effect of factors on the distance from the SP to great vascular bifurcations.

**Results:**

There were 20 cases of IIV variation (20.2%). VW was 28.8 ± 12.4 mm in the variant group and 39.6 ± 12.6 mm in the standard group (*p* = 0.001). In the ANCOVA model, IIV variations affected VW (coefficient, –11.8; 95% confidence interval [CI], –18.4 to –5.08, *p* < 0.001). Multivariate analysis revealed that the aorta–SP distance decreased with age (coefficient, −0.44; 95% CI, −0.77 to −0.11, *p* = 0.009).

**Conclusions:**

One in five women has a vascular variant at the SP that restricts the “safe” zone of fixation to < 3 cm.

**Supplementary Information:**

The online version contains supplementary material available at 10.1007/s00192-023-05681-4.

## Introduction

The sacral promontory (SP) is an important landmark in sacrocolpopexy for repairing pelvic organ prolapse (POP) [1]. Knowledge of the anatomy of the presacral space is important because there are large vessels in this space that may be accidentally injured while exposing the anterior longitudinal ligament (ALL) for sacrocolpopexy procedures, leading to life-threatening hemorrhage [2]. The left common iliac vein (CIV) is the most commonly major vessel injured while exposing the ALL in the SP region [3]. To our knowledge, no study has used three-dimensional computed tomography (3DCTA) to preoperatively evaluate the internal iliac vein (IIV) relative to the anterior SP and assess whether the vasculature affects the choice of mesh anchoring site above or below the SP. A recent retrospective cohort study reported [4] that the IIV was divided into standard and variant IIV, which were further subdivided into different subtypes. The anatomical structure of the iliac vein was defined as standard if it met the following two conditions. First, each CIV was formed by the ipsilateral external iliac vein (EIV) and the low IIV. Second, the left and right CIV together formed the right inferior vena cava (IVC), which was the most common anatomical type of iliac vein. There are several reports on IIV variations [4–6], but most of the studies are based on cadaveric samples or two-dimensional CT images, which do not reflect the in vivo anatomy [7–9]. Some studies have shown that the vessel location from all directions reconstructed using 3D models of pelvic vessels provides useful information for the analysis of individual anatomical structures. However, a limitation of these studies is the small number of cases [10] and the lack of consistency between the findings and the actual intraoperative findings [11]. To provide surgeons with detailed and reliable anatomical knowledge, 3DCTA should be used to compare standard and variant IIV to analyze the extent of possible dissection without affecting vessels before the SP [11], including the width of the vascular window (VW), the medial-most vessel in the VW on either side [12], and the distance from the SP to the aorta or IVC bifurcation [2]. We hypothesized that patients with IIV variations would have a narrow VW.

## Materials and methods

### Study design

This prospective study, conducted at Hokusuikai Kinen Hospital in Mito, Ibaraki, was approved by the institutional review board (approval number: 2022-078). The informed consent process included providing information about the purpose of this study, the benefits and risks of contrast-enhanced CT imaging, and the hospital’s response if there were complications, along with details of the surgical procedure. Patients who provided consent were included in the study. Patients were enrolled from July 2022 to April 2023. VW was defined as the free vascular area for the anterior part of the SP. The primary endpoint was difference in the width of the VW anterior to SP between the standard and variant IIV groups on preoperative 3DCTA. The secondary endpoint was the difference between the two groups adjusted for age, body mass index (BMI), hypertension, and diabetes using an analysis of covariance (ANCOVA) model.

### Data collection

Patients were eligible for enrollment if they were scheduled for laparoscopic sacrocolpopexy (LSC) for POP treatment and were aged ≥18 years. The exclusion criterion was a contraindication to CT. Patients with a history of pelvic radiation or spinal surgery involving the lumbar or sacral spine were also excluded. Concomitant procedures, such as vaginal and laparoscopic posterior repairs, supracervical hysterectomy, and uterine preservation, were not a reason for patient exclusion.

Patients provided consent in the clinic before undergoing LSC. A surgeon (H.S.) obtained signed informed consent from all the participants. All procedures were conducted by a trained urologist (H.S.) following our operative procedure [13]. The mesh was sutured to the ALL at or around S1.

Demographic and clinical variables, including preoperative Pelvic Organ Quantification results and surgical details, were collected from medical charts and surgical records.

All patients underwent preoperative 3DCTA using the following protocol. All CT examinations were performed using different machines, such as 80-channel (Acqillion Serve 80, Canon Medical Systems, Tochigi, Japan) CT systems. Compared with conventional models, these models have advanced intelligence, in which reconstruction technology selectively removes noise and maintains resolution, contributing to reduced radiation exposure in CT examinations and providing high-quality images. Omnipaque 300 (GE Healthcare, Tokyo, Japan) was administered intravenously at 2 ml/s. The scanning parameters included tube voltage/current 120Kv/75–190 mA, rotation time 0.75 s, pitch 0.915, collimation 0.5 mm, and section thickness/reconstruction interval 5 mm. 3D reconstructions were generated using the imaging software SYNAPSE VINCENT^®^ (Fujifilm Medical Co., Tokyo, Japan). An image from the umbilicus to the SP was created to account for the laparoscopic field of view.

**Fig. 1:**
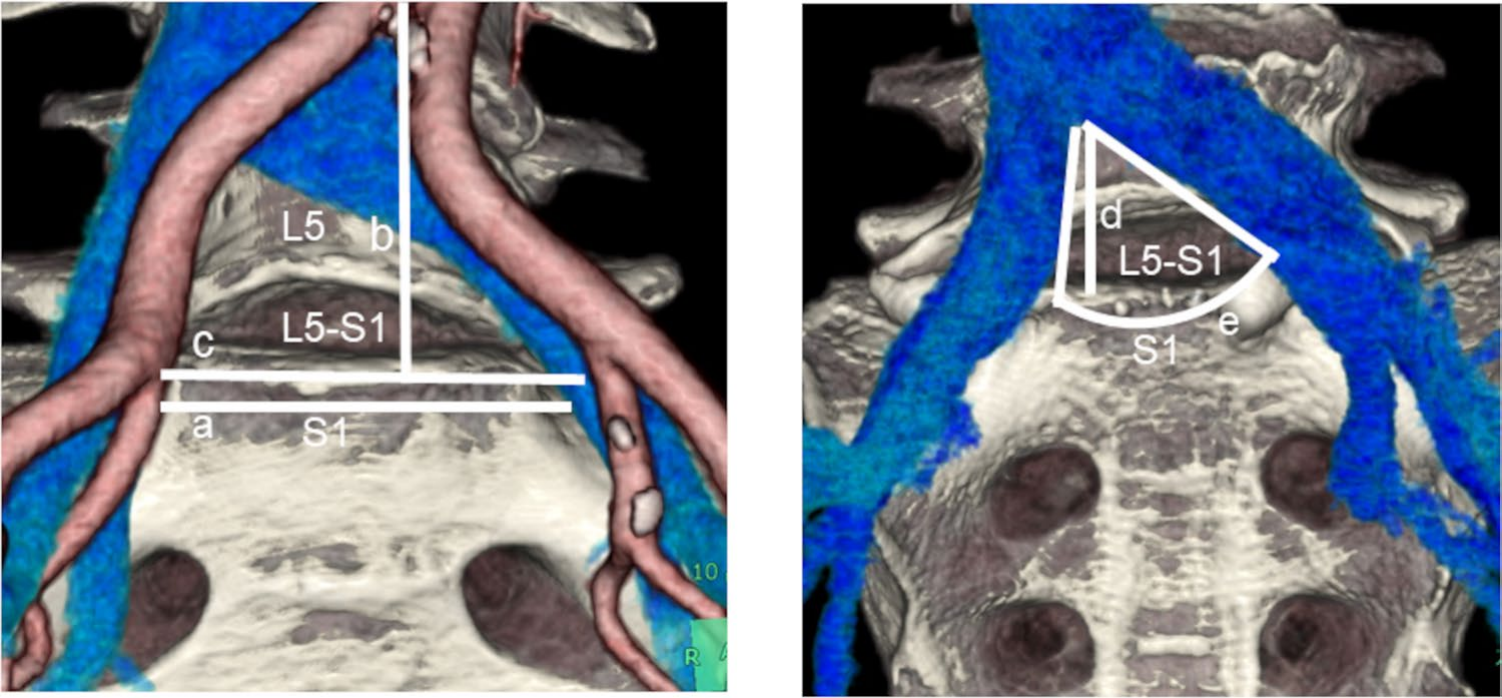
Measurement parameters of the internal iliac vein anterior to the sacral promontory. **a** VW; **b** bifurcation of the aortic distance; **c** width of the SP; **d** bifurcation of the IVC distance; **e** angle of IVC bifurcation. *IVC* inferior vena cava, *SP* sacral promontory, *VW* vascular window

The following anatomical parameters were determined (Fig 1):

1. The VW at SP in millimeters was defined as the distance between the medial-most iliac vascular structure (artery or vein) on the right and the medial-most iliac vascular structure (artery or vein) on the left, corresponding to the “free vascular” area at the anterior part of the SP (Fig. 1a).
2. The distance of the aortic bifurcation: the vertical distance from the bifurcation of the aorta to the SP Fig. 1b.
3. The SP width: the width of the upper surface of the first sacral vertebra (Fig. 1c).
4. The distance of the IVC bifurcation: the vertical distance from the bifurcation of the IVC to the SP Fig. 1d.
5. The angle of IVC bifurcation: the angle between the confluence of the left and right CIV in cases of IIV variation; the default angle is that between the confluence of the variant IIV and the other CIV or the combined angle of the confluence of the bilateral variant IIV (Fig. 1e).

All measurements were obtained twice, once by the participating radiologist (M.K.) and once by the urologist (H.S.). The average of these measurements was used for the data analysis. The SP was defined as the superior-most point on the anterior surface of the S1 vertebra. This study focused on the great vessels and the IIV. Measurements of the VW in this study did not include the ureter, mid-sacral vessels, or nerves.

Sample sizes of 79 and 20 patients in the standard and variant groups respectively provided a post hoc power of 93% to detect differences in a mean of 10 mm, assuming a common standard deviation (SD) of 12, based on a type 1 error rate of 5%. Between-group comparisons were performed using the Chi-squared test for categorical variables and Student’s *t* test for continuous variables. Continuous variables are expressed as mean ± SD, 95% confidence interval (CI), or median and interquartile range (IQR). Categorical variables are expressed as frequencies and percentages. The coefficient and two-sided 95% CI of the difference between the standard and variant groups were calculated using an ANCOVA model adjusted for group, age, BMI, hypertension status, and diabetes status. Univariate and multivariate regression analyses were used to calculate the correlation between the dependent variable (bifurcation of the aorta or IVC distance) and independent continuous variables, such as age and BMI. All tests were two-sided, and significance was set at *p* < 0.05. All statistical analyses were performed using R (R Foundation for Statistical Computing, Vienna, Austria) and EZR (Saitama Medical Center, Jichi Medical University, Saitama, Japan). We performed a sensitivity analysis, assessed additional variables such as the presence or absence of iliac artery tortuosity, and constructed an ANCOVA model to calculate the coefficient and 95% CI.

## Results

**Fig. 2:**
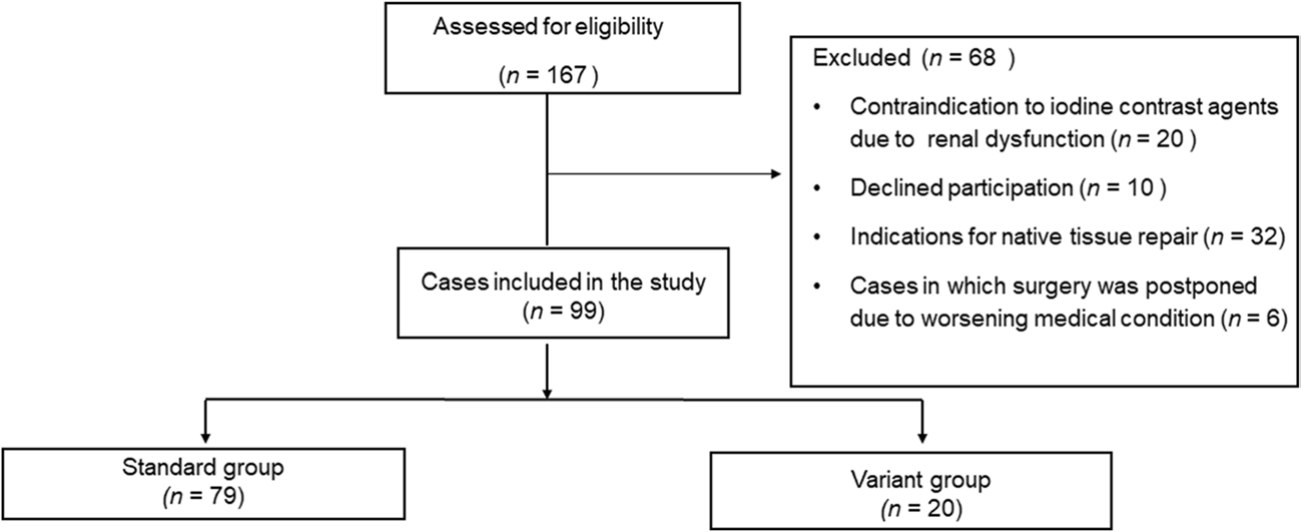
Patient distribution as per study design

A total of 167 consecutive patients with POP who underwent 3DCTA from July 2022 to April 2023 were enrolled in the study. Patients were excluded for the following reasons: refusal to consent to the study (*n* = 10), contraindication to iodine contrast agents owing to renal dysfunction (*n* = 20), indications for native tissue repair (*n* = 32), and surgery postponement due to a worsening medical history (*n* = 6). Finally, 99 women were included in the analyses (Fig. 2). Table 1 summarizes the baseline demographic characteristics of the study group. Weight and BMI were significantly lower in the variant group than in the standard group (53.2 kg vs 57.2 kg, *p* = 0.041 and 23.3 kg/m^2^ vs 25 kg/m^2^, *p* = 0.024 respectively). The variant group had a significantly lower rate of hypertension than the standard group (9% vs 56%; *p* = 0.037). Table 2 shows the VW in front of the SP measurements and other parameters in the two groups and the classification of IIV variants. The VW of the SP on 3DCTA was significantly narrower in the variant than in the standard group (28.8 mm vs 39.6 mm, *p* = 0.001). The distance from the bifurcation of the IVC to the SP was also shorter in the variant group than in the standard group (19.1 mm vs 31.5 mm, *p* = 0.002).

**Table 1:** Baseline demographic and operative characteristics of the study groups

Variable	Standard (*n* = 79)	Variant (*n* = 20)	*p*
Age, years	73.3 ± 6.9	74.2 ± 7.3	0.61
Height (cm)	151.2 ± 6.2	151.2 ± 6.0	0.98
Weight (kg)	57.2 ± 7.9	53.2 ± 7.2	0.041*
BMI, kg/m^2^	25 ± 3.1	23.3 ± 2.9	0.024*
Parity	2 (2–3)	2 (2–2)	0.39
Former tobacco use	6 (7.6)	0	0.34
Hypertension	56 (70.9)	9 (45)	0.037*
Diabetes	23 (29.1)	2 (10)	0.091
Previous prolapse repair	3 (3.8)	1 (5.0)	1.00
Previous hysterectomy	13 (16.5)	2 (10)	0.73
OT, min	100 (89.5–120)	101 (85–113)	0.40
EBL, mL	10 (5.0–20)	10 (8.8–10)	0.55
POP-Q stage II	1 (1.3)	0	0.67
POP-Q stage III	56 (70.9)	13 (65)	
POP-Q stage IV	22 (27.8)	7 (35)	
SCH	35 (44.3)	14 (70)	0.048*
UP	31 (39.2)	4 (20)	0.12

Data are shown as numbers (%), means (±SD), or medians (IQR)

*BMI* body mass index, *EBL* estimated blood loss, *IQR* interquartile range, *OT* operative time, *POP-Q* Pelvic Organ Prolapse Quantification, *SCH* supracervical hysterectomy, *UP* uterine preservation, * Statistically significant difference at *p* < 0.05

**Table 2:** Measurement of SP vascular windows and other parameters between the two groups, and classification of internal iliac vein variants

Variable	Standard (*n* = 79)	Variant (*n* = 20)	95% CI	*p*
VW (mm)	39.6 ± 12.6	28.8 ± 12.4	4.54 to 17.0	0.001*
Bifurcation of IVC angle (°)	76 ± 22.5	84.1 ± 16.4	–19.3 to 2.97	0.15
Bifurcation of aorta distance (mm)	49.1 ± 12.2	48.1 ± 10.6	–4.96 to 6.86	0.75
Bifurcation of IVC distance (mm)	31.5 ± 16.1	19.1 ± 7.5	4.79 to 19.9	0.002*
Width of SP (mm)	57.5 ± 7.5	57.2 ± 7.6	–3.53 to 3.95	0.91
Left borders of VW				
LCIV	59 (78.7)	11 (57.9)		0.003*
LCIA	4 (5.3)	0		
LIIA	9 (12.0)	1 (5.3)		
LIIV	3 (4.0)	6 (31.6)		
LIIV visceral branch	0	1 (5.3)		
Right borders of VW				< 0.001*
RIIA	54 (72.0)	5 (26.3)		
RCIA	15 (20.0)	1 (5.3)		
RCIV	2 (2.7)	2 (10.5)		
RIIV	3 (4.0)	9 (47.4)		
RIIV parietal branch	1 (1.3)	0		
REIA	0	2 (10.5)		
Iliac artery tortuosity				
No	63 (79.7)	15 (75.0)		0.76
Yes	16 (20.3)	5 (25.0)		
Type of				
Standard IIV 1	79			
Variant IIV 2a		0		
2b		2 (2.0)		
3a		4 (4.0)		
3b		1 (1.0)		
4a		2 (2.0)		
4b		4 (4.0)		
5a		3 (3.0)		
5b		0		
5c		0		
5d		0		
Others		4 (4.0)		

Data are shown as numbers (%), means (±SD), or medians (IQR)

Type 1, normal iliac venous connection; type 2, high joining of the IIV to the ipsilateral EIV (2a: right IIV variation; 2b: left IIV variation); type 3, IIV joining to the contralateral CIV (3a: right IIV variation; 3b: left IIV variation); type 4, IIV forming a common trunk (4a: absence of communicating branches between the IIV and ipsilateral EIV; 4b: presence of communicating branches between the IIV and ipsilateral EIV); type 5, connection between the IIV and contralateral CIV or between the bilateral IIV (5a: presence of communicating branches between the right IIV (RIIV) and left CIV (LCIV); 5b: presence of communicating branches between the left IIV (LIIV) and right CIV (RCIV); 5c: presence of communicating branches between the RIIV and the upper end of the LIIV; 5d: presence of communicating branches between the LIIV and the upper end of the RIIV). Patients without these variants were classified as having other IIV variants

*CIV* common iliac vein, *EIV* external iliac vein, *IIV* internal iliac vein, *IVC* inferior vena cava, *LCIA* left common iliac artery, *LCIV* left common internal vein, *LIIA* left internal iliac artery, *LIIV* left internal iliac vein, *RCIA* right common iliac artery, *RCIV* right common iliac vein, *REIA* right external iliac artery, *RIIV* right internal iliac vein, *SP* sacral promontory, *VW* vascular window, * Statistically significant difference at *p* < 0.05

**Fig. 3:**
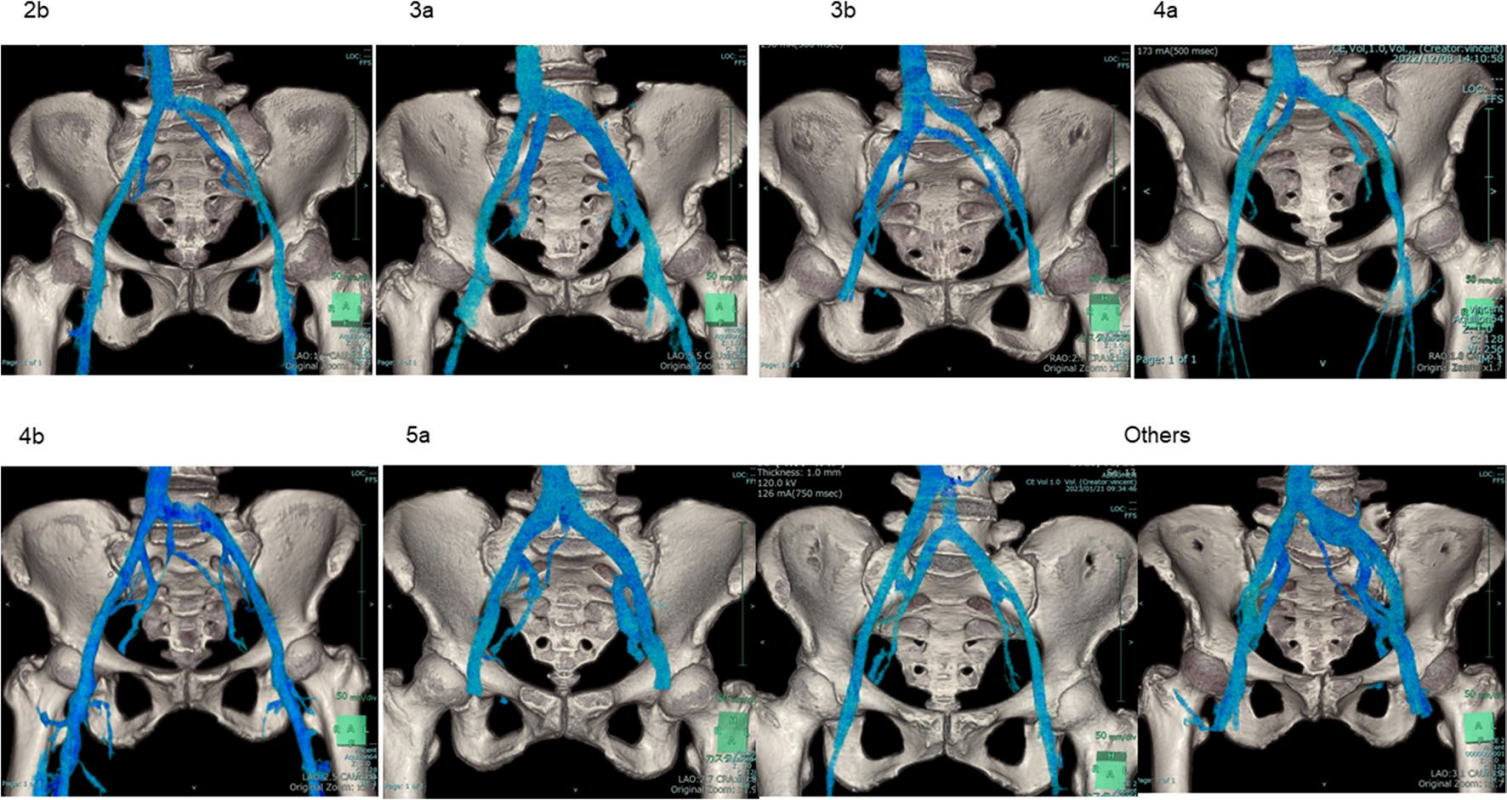
Internal iliac vein classification

The aortic and IVC bifurcations were completely caudal to the SP in three cases (3.0%); in those cases, the VW was 0 mm. Vessels on the left border of the VW in the variant group had a lower proportion of LCIV than those in the standard group (11 cases [57.9%] vs 59 cases [78.7%], *p* = 0.003). Vessels on the right border of the VW significantly differed between the standard and variant groups, with the right internal iliac artery being the most common vessel in 54 patients (72.0%) in the standard group and five patients (26.3%) in the variant group (*p* < 0.001). Twenty patients (20.2%) had an IIV variant. The variation rate according to type was 3a (4.0%), 4b (4.0%), > 5a (3.0%), > 2b (2.0%), 4a (2.0%), and > 3b (1.0%). The other variant type accounted for 4.0% (Fig. 3). Table 3 shows the adjustments for VW according to the ANCOVA model. The VW of the variant against the standard group had an adjusted coefficient of –11.8 (95% CI, –18.4 to –5.08; *p* < 0.001).

**Table 3:** Factors associated with vascular window: analysis of covariance model of patients who underwent three-dimensional computed tomography angiography before laparoscopic sacrocolpopexy

Characteristics	Coefficient	95% CI	*p*
Age, years	0.24	–0.12 to 0.60	0.19
BMI	0.14	–0.69 to 0.97	0.73
Group	–11.8	–18.4 to –5.08	< 0.001*
Diabetes	–1.36	–7.29 to 4.57	0.65
Hypertension	–3.14	–8.82 to 2.53	0.27

*BMI* body mass index, *CI* confidence interval, * Statistically significant difference at *p* < 0.05

Supplementary Table 1 shows the univariate and multivariate regression models predicting aortic and IVC bifurcation to SP distance. In the univariate regression analysis, age (*p* = 0.011) was significantly associated with bifurcation of aortic distance, and BMI (*p* = 0.003) was significantly associated with bifurcation of IVC distance. To estimate the distance from the aortic bifurcation to the SP, age and BMI were examined using multivariate regression analysis. Age as an independent variable correlated significantly with aortic bifurcation distance in the multivariate analysis, with a coefficient of −0.44 (95% CI, −0.77 to −0.11; *p* = 0.009).

Supplementary Table 2 presents the ANCOVA model adjusted for iliac artery tortuosity in addition to the explanatory variable. The difference in VW between the variant and standard groups had an adjusted coefficient of –11.2 (95% CI, –17.9 to –4.57; *p* = 0.001).

## Discussion

This study investigated the preoperative 3DCTA images of patients scheduled for LSC to examine IIV variants. The VW anterior to the SP was measured by comparing groups with or without the variants. The variant group had a significantly smaller VW than the other group, and the ANCOVA model was used to analyze VW adjusted for sociodemographic factors and comorbidities. There was a significant difference between the groups with and without IIV variants, excluding the effect of covariates on the VW. This study also showed that the distance from the SP to the aortic bifurcation decreased with age. Accordingly, the results were consistent with the stated hypothesis.

Previous studies have shown varying results regarding the measurement of the VW in front of the SP in sacrocolpopexy or anterior lumbar interbody fusion due to differences in race, measurement methods, and sex [11, 14, 15]. As expected, the presence of IIV variants in ALL of the SP seems common (20.2%) [5, 11]. If the left or right IIV flows into the contralateral CIV (types 3 and 5a) or if the left and right IIV merge to form a common trunk and their common trunk is the confluence of the left or right CIV or both sides (type 4), then the VW would be considered narrow [4, 11].

Furthermore, the distance from the SP to the IVC bifurcation was also significantly shorter in the variant group than that in the standard group, suggesting that the LCIV and SP were relatively close. The LCIV, the site of major vessel injury during LSC [16–18], requires careful ALL dissection and retraction. In this study, the ANCOVA model showed a significant difference in VW between the variant and standard groups. The VW in the variant group was narrower than that in the standard group, which should be considered during surgery. The surgeon was able to visualize the relationship between the vascular structures and the SP from all directions with 3DCTA and clearly understood the vascular structures. As a result, careful dissection could be performed intraoperatively.

The distance between the aortic bifurcation and SP decreased with age [2, 10, 19, 20]. This may be biologically plausible because the aging of the lumbar spine is accompanied by disk compression and loss of bone mineral density, resulting in decreased height [10]. Moreover, aging may result in calcium accumulation (a measure of atherosclerosis) and increased aortic diameter (a possible measure of elastin loss) [19–22], which may contribute to a downward shift of the aorta.

This study has several limitations. First, blinding was not incorporated into the analysis. The corresponding author (H.S.) and coauthors, M.K. and S.O., reviewed all the preoperative 3DCTA images, and a rigorous review of the accuracy of the imaging diagnosis was not conducted. Second, ALL dissection in the VW was limited to the area where suture fixation of the mesh could be safely performed. Therefore, the vascular structures around the ALL (e.g., the middle sacral artery and vein) were not completely confirmed intraoperatively, and there may have been cases in which the preoperative diagnosis was inaccurate owing to differences between the preoperative 3DCTA findings and the actual anatomical structures. However, the extent of the ALL dissection allowed suturing; moreover, unnecessary dissection carries the risk of vascular injury [18]. If the anatomy of the IIV could not be confirmed intraoperatively, caution may be warranted in women with a low BMI or retroperitoneal adhesions after laparotomy [12].

Third, we employed CT, and a limitation of this modality is that structures with high attenuation, such as bones, may mask the target anatomy [23]. In the present study, delineating the variant vessels was difficult in some patients, who had low retroperitoneal fat, owing to the proximity of some bones to the vessels.

A strength of this study is that all procedures were completed without vascular injury.

In summary, in this prospective cohort study we demonstrated that 1 in 5 women undergoing LSC had a vascular variant that restricts the safe VW for promontory dissection and fixation to a distance of 28 mm. Older women with a low BMI had a significantly higher risk of a vascular variant and consideration for preoperative 3DCTA may be indicated in these patients to avoid vascular injury during surgery.

### Supplementary information

Below is the link to the electronic supplementary material.

Supplementary figure 1. Preoperative 3DCTA and intraoperative findings showing iliac artery tortuosity with atherosclerosis and a narrow VW. *MSV* midsacral vein, *RIIA* right internal iliac artery, *RIIV* right internal iliac vein, *SP* sacral promontory (PNG 142 kb)

High Resolution Image (TIF 1059 kb)

Supplementary file2 (PDF 103 KB)

## Data Availability

The data that support the findings of this study are not publicly available in order to protect the identity of the patients. However, the data can be made available upon reasonable request from the corresponding author, HS.
