# Supplementary figures and images for: Iliac vein variation in the sacral promontory on three-dimensional computed tomography angiography: a prospective observational study before laparoscopic sacrocolpopexy
Source: Int Urogynecol J. 2023 Nov 24;35(1):167–73. doi: 10.1007/s00192-023-05681-4 (PMC10810968; doi:10.1007/s00192-023-05681-4)

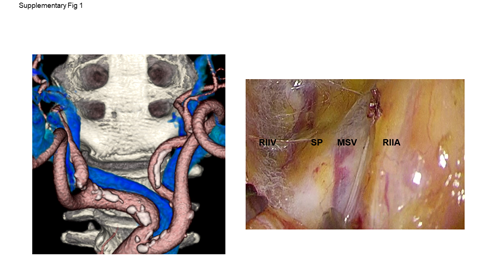

Supplement: Supplementary file 1 — Preoperative 3DCTA and intraoperative findings showing iliac artery tortuosity with atherosclerosis and a narrow VW. MSV midsacral vein, RIIA right internal iliac artery, RIIV right internal iliac vein, SP sacral promontory (PNG 142 kb) [file 192_2023_5681_Fig4_ESM.png]

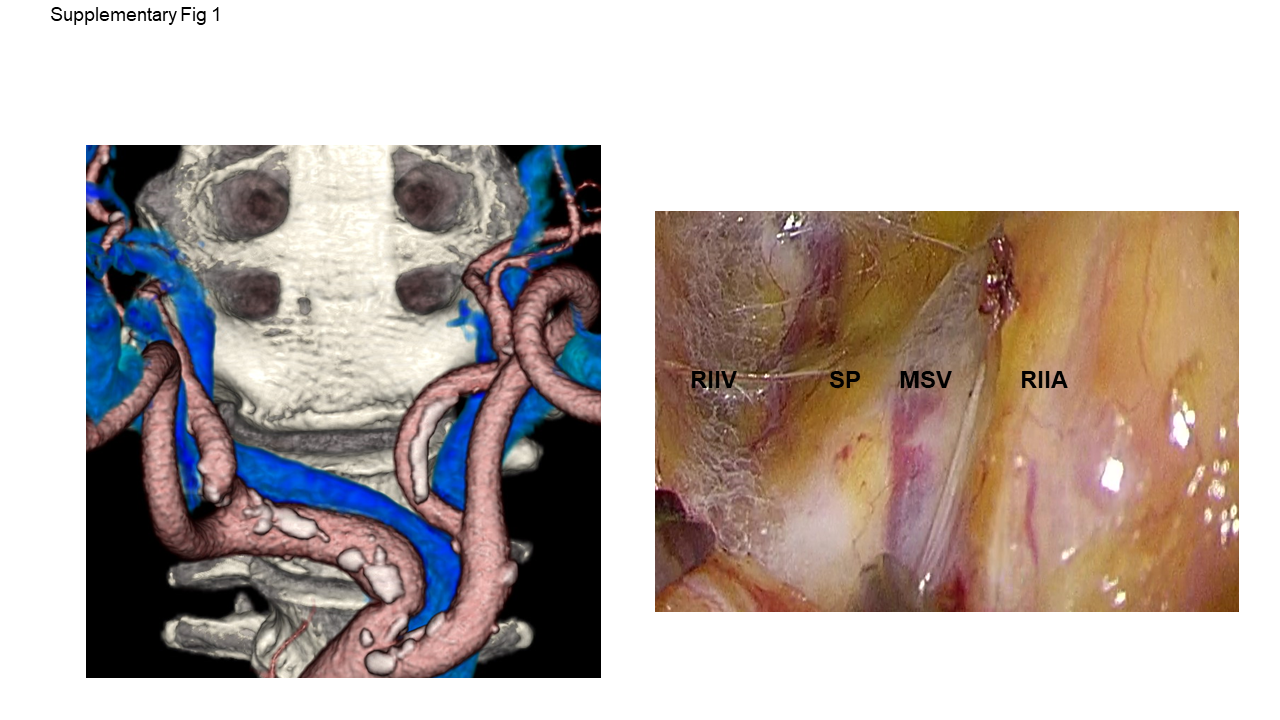

Supplement: Supplementary file 2 — High Resolution Image (TIF 1059 kb) [file 192_2023_5681_MOESM1_ESM.tif]
