# Supplementary material for: Iliac vein variation in the sacral promontory on three-dimensional computed tomography angiography: a prospective observational study before laparoscopic sacrocolpopexy
Source: Int Urogynecol J. 2023 Nov 24;35(1):167–73. doi: 10.1007/s00192-023-05681-4 (PMC10810968; doi:10.1007/s00192-023-05681-4)
Supplement: Supplementary file 3 — Supplementary file2 (PDF 103 KB) [file 192_2023_5681_MOESM2_ESM.pdf]

Supplementary Table 1. Univariable and multivariable regression models for predictors of aortic and IVC bifurcation to SP distance

Bifurcation of aortic distance

	Univariable analysis		Multivariate analysis	
Factors	Coefficient (95% CI)	*P*		*P*
Age, y	-0.43 (-0.76 – -0.10)	0.011*	-0.44 (-0.77 – -0.11)	0.009*
BMI	0.53 (-0.23 – 1.29)	0.17	0.56 (-0.18 – 1.30)	0.14

Bifurcation of the IVC distance

	Univariate analysis		Multivariate analysis	
Factors	Coefficient (95% CI)	*P*		*P*
Age, y	0.028 (-0.42 – 0.48)	0.90	0.003 (-0.43 – 0.44)	0.98
BMI	1.51 (0.54 – 2.49)	0.003*	1.51 (0.54 – 2.49)	0.003*

BMI, body mass index; CI, confidence interval; IVC, inferior vena cava; SP, sacral promontory

Supplementary Table 2. AVCOVA model adjusted by adding iliac artery tortuosity

Characteristics	Coefficient	95% CI	P
Age, y	0.29	–0.085 to 0.66	0.13
BMI	0.33	–0.53 to 1.20	0.45
Group	–11.2	–17.9 to –4.57	0.001*
Diabetes	–1.30	–7.18 to 4.58	0.66
Hypertension	–2.97	–8.6 to 2.66	0.30
Iliac artery tortuosity	–4.99	–11.2 to 1.25	0.12

ANCOVA, analysis of covariance; BMI, body mass index; CI, confidence interval.
